# Metabolic Profiling and Metabolite Correlation Network Analysis Reveal That *Fusarium solani* Induces Differential Metabolic Responses in *Lotus japonicus* and *Lotus tenuis* against Severe Phosphate Starvation

**DOI:** 10.3390/jof7090765

**Published:** 2021-09-16

**Authors:** Amira Susana Nieva, Fernando Matías Romero, Alexander Erban, Pedro Carrasco, Oscar Adolfo Ruiz, Joachim Kopka

**Affiliations:** 1 Max Planck Institute of Molecular Plant Physiology (MPI-MP), Am Mühlenberg 1, 14476 Potsdam, Germany; Erban@mpimp-golm.mpg.de (A.E.); Kopka@mpimp-golm.mpg.de (J.K.); 2 Postdoctoral Fellow—Deutscher Akademischer Austauschdienst (DAAD), Kennedyallee 50, 53175 Bonn, Germany; 3 Instituto Tecnológico de Chascomús (INTECH), Universidad Nacional de San Martin (UNSAM), Av. Intendente Marino Km 8.2, Chascomús 7130, Argentina; mromero@intech.gov.ar (F.M.R.); ruiz@intech.gov.ar (O.A.R.); 4 Institut de Biotecnològia i Biomedicina (BIOTECMED), Universitat de València, Av. Doctor Moliner 50, 46100 Burjassot, Spain; pedro.carrasco@uv.es

**Keywords:** *Fusarium solani*, *Lotus* spp., phosphate starvation, correlation network analysis, metabolomics

## Abstract

Root fungal endophytes are essential mediators of plant nutrition under mild stress conditions. However, variations in the rhizosphere environment, such as nutrient depletion, could result in a stressful situation for both partners, shifting mutualistic to nonconvenient interactions. Mycorrhizal fungi and dark septate endophytes (DSEs) have demonstrated their ability to facilitate phosphate (Pi) acquisition. However, few studies have investigated other plant–fungal interactions that take place in the root environment with regard to phosphate nutrition. In the present research work, we aimed to analyze the effect of extreme Pi starvation and the fungal endophyte *Fusarium solani* on the model *Lotus japonicus* and the crop *L. tenuis.* We conducted metabolomics analysis based on gas chromatography-mass spectrometry (GC-MS) on plant tissues under optimal conditions, severe Pi starvation and *F.solani* presence. By combining statistical and correlation network analysis strategies, we demonstrated the differential outcomes of the two plant species against the combination of treatments. The combination of nutritional stress and *Fusarium* presence activated significant modifications in the metabolism of *L. japonicus* affecting the levels of sugars, polyols and some amino acids. Our results display potential markers for further inspection of the factors related to plant nutrition and plant–fungal interactions.

## 1. Introduction

*Lotus* spp. belong to the *Fabaceae* family, which contains approximately 130 species. *Lotus* legumes have the ability to tolerate different biotic and abiotic stresses, and some of them are forage crops in constrained areas that have limitations for traditional agriculture use due to the restrictive soil conditions [1]. *Lotus tenuis* is a legume used as a forage resource in the Flooding Pampa region (Buenos Aires, Argentina). This region is mainly characterized by flooding, high salinity and high pH values, which lead to the low availability of nutrients such as phosphorus and iron in the soil. *L. tenuis* can grow and fully develop in these restrictive conditions; thus, it has been successfully naturalized in the Flooding Pampa region [1,2,3]. Research performed on *L. tenuis* has been accompanied by research on the model *L. japonicus* [4]. Both species have been used to characterize the response of legumes to biotic and abiotic stresses. In particular, abiotic stress responses have been extensively studied, including that on a larger panel of *Lotus* species [5,6,7,8,9,10,11].

The introduction of *L. tenuis* in the Flooding Pampa region has modified the soil fungal diversity and the soil environment. Mycorrhizal sporulation and viability dropped due to the introduction of *L. tenuis* and the agricultural practices applied during its cultivation [12]. The increase in the richness of *Fusarium* spp. in the constrained soils of the Flooding Pampa region has been associated with the presence of *L. tenuis* [13]. The fungal endophyte *Fusarium solani 142L52B*, which is isolated from the roots of *L. tenuis* growing in conditions of salinity, alkalinity and low phosphate (Pi), manages to infect the tissues of *L. tenuis* and *L. japonicus*, with differential effects based on the *Lotus* species [14]. This endophyte can solubilize Pi in vitro and tolerate salinity and alkalinity. Nevertheless, it was not able to improve the total phosphorus content in the shoots of *Lotus* spp. under optimal growth conditions [14]. Fungal endophytes constitute a wide group of organisms with the ability to colonize plant tissues without producing damage or an appearance of symptoms. The study of fungal endophytes involves ecological aspects regarding the outcome in plant-endophyte systems. In this sense, different theories have been proposed to explain the variability in the performance of these interactions [15,16].

Phosphorus is the second most important mineral nutrient essential for plant growth, and low Pi availability severely limits crop and pasture growth [17]. The relationship between mycorrhizal fungi and the improvement of Pi uptake has been extensively studied in several plant species [18]. The effect of mycorrhizal fungi on *Lotus* species under Pi starvation conditions has also been described [19,20], but information is scarce regarding the improvement of phosphorus uptake, as Pi is mediated by other mutualistic plant–fungal associations [21]. Other nonmycorrhizal fungi, such as endophytes belonging to the genus *Neotyphodium* [22] and dark septate endophytes (DSEs) [23], assist their hosts in obtaining Pi nutrition. Motivated by this evidence, we explored the effect of a *Fusarium* endophyte as a potential symbiotic partner on the Pi status of their plant hosts.

The partitioning of carbon resources constitutes an important component of plant–fungal interactions. The carbon requirements of the fungal partner can enhance the photosynthesis rate to increase photosynthesis-derived compounds [24]. Plant-derived sucrose is a key element provided as a carbon resource for fungi that establish symbiotic [25] and pathogenic interactions [26]. Our previous study demonstrated the involvement of sugars in the interaction between *Lotus* spp. and *F. solani* [14]. Moreover, there is evidence regarding Pi interaction and sugar metabolism in other plant systems [27,28,29].

In addition to studying conventional means to analyze metabolic changes, we aimed to analyze the fluctuations in the metabolic system that induce variability in metabolite concentrations and thereby generate patterns of correlations among metabolite abundances [30]. Such correlations can exist even between metabolites that do not share the same metabolic pathway [30,31]. Correlation networks constitute a powerful tool to investigate the relationships between metabolites. The elements of such correlation networks are nodes, which represent metabolites, and edges, which represent correlations or interactions between metabolites. *Hubs* in a network are those nodes that are much more connected than average or typical nodes. *Hubs* are consequently assumed to play crucial biological roles [32]. Considering the correlation network approach, the detection of patterns among metabolite interactions may change in response to the adaptation or acclimation of plant metabolism to environmental conditions. Such changes in interactions are typically not revealed by analyses of individual metabolites.

Evidence from the fungal community in soils of the Flooding Pampa has built a framework for research on plant–fungal interactions in this constrained environment. The premise that relates *Fusarium* diversity and anthropogenic introduction of *L. tenuis* is still unclear, but it is reasonable to assume that the plant–fungal interactions are in status nascendi and that *Fusarium* organisms may play significant roles in the ecosystem colonized by *Lotus* species. In the current study, we describe the *Lotus* spp.–*Fusarium solani* interaction under extreme Pi starvation. We hypothesize that Pi is a driving factor for establishing the fungal endophyte *F. solani* in *Lotus* species. To assess the effect of *F. solani*, we inoculated roots of the model species *L. japonicus* and of the forage crop *L. tenuis* with the endophyte and explored interactions with Pi nutrition in root and shoot tissue by comparing the Pi sufficiency to Pi starvation in inoculated and noninoculated plants. We profiled and analyzed the primary metabolism by conventional statistical approaches and took a step beyond by including a comparative correlation network approach as a strategy to detect changes in interactions between central metabolites in response to our experimentally defined conditions.

## 2. Materials and Methods

### 2.1. Plant Material, Inoculation, Growth Conditions and Sampling

Seeds of *L. japonicus* (ecotype Gifu B-129) and *L. tenuis* (cv. “Nahuel”) were scarified with sulfuric acid for 3 min and washed 10 times with distilled water. Furthermore, the sample seeds were surface disinfected with 5% sodium hypochlorite and washed with sterile distilled water. The scarified-disinfected seeds were incubated overnight in sterile distilled water until imbibition. The seeds were placed in Petri dishes containing an agar-water medium (0.5% *w*/*v*) and cultivated in growth chamber under a 16/8 h photoperiod at 24/19 °C (day/night), a light intensity of 240 mmol^−2^ s^−1^ and 60% humidity for 10 days. The fungal endophyte *F. solani strain 142L52B* (in the following *Fsol*), which was isolated from roots of *L. tenuis* growing in constrained soils [14], was used to inoculate roots of *L. japonicus* and *L. tenuis* under experimental conditions.

The fungal strain was cultivated in a Sabouraud agar medium (SAM) [33] for 12 days at 28 °C in dark conditions. Solid SAM (approximately 1 cm^−2^) plugs containing *Fsol* mycelia were used as inoculants. The inoculation of *Lotus* spp. roots with *Fsol* was carried out during the seedling transplant. Seedlings were transferred to pots containing a quartz-sand:perlite substrate (proportion 3:1). Each seedling was inoculated with SAM plugs containing *Fsol* mycelia (FUS+). Control inoculation was performed with sterile plugs of fresh media (noninoculated). The inoculum was placed at 1 cm depth in each pot. Simultaneously, sufficient Pi and Pi starvation treatments were performed using modified Evans solution [34] containing 0.1 mM KH_2_PO_4_ and 0.1 mM K_2_HPO_4_ as Pi sources or an Evans solution without the Pi fraction. Each combination between species and inoculation was performed under optimal Pi conditions (P+) or Pi starvation (P-) using the above-indicated nutrient solutions.

The final set of experimental treatments of *L. japonicus* and *L. tenuis* included the following combinations: Control (noninoculated, optimal Pi), FUS+ (inoculated, optimal Pi), P- (noninoculated, Pi starvation) and FUS+P- (inoculated, Pi starvation). The experiment was carried out with 10 biological replicates per treatment combination. Plants were harvested 32 days postinoculation and following Pi treatment. Shoots and roots were separated, frozen, ground in liquid nitrogen and stored at −80 °C until analysis.

### 2.2. In Vitro Cultivation of Fusarium solani 142L52B and Sampling of Mycelia and Secreted Compounds

To determine the compounds secreted by *Fsol* and the compounds accumulated by the mycelial cells, we analyzed polar metabolites from mycelia and the supernatant medium produced by *Fsol* under in vitro conditions. Cultivation of *Fsol* was performed by inoculating one plug of approximately 1 cm^−2^ in 50 mL of Sabouraud broth medium (SAB) incubated on a shaker for 7 days at 28 °C. Fresh SAB was used as the control. The experiment was conducted in five biological replicates. After 7 days, mycelia were separated from the supernatant medium by filtration and ground to a fine powder in liquid nitrogen. The mycelia and supernatant were stored at −80 °C until metabolite extraction.

### 2.3. Metabolite Extraction and Profiling of Plant Tissues, Mycelia and Fungal Supernatant

The extraction of polar metabolites from *Lotus* tissues was conducted using methanol:chloroform:water liquid-phase extraction [35]. The general extraction procedures were carried out as reported by Erban et al. [36]. An extraction solution containing 300 µL of methanol and 30 µL of a ^13^C_6_-sorbitol stock solution (0.2 mg/mL in distilled water) was added to 200 mg of finely ground fresh material. The mixture was heated at 70 °C for 15 min, followed by the addition of 200 µL of chloroform and incubation at 37 °C for an additional 5 min. Polar phase separation was induced by adding 400 µL of distilled water and centrifugation at 20,000× *g* for 5 min. An aliquot of 160 µL in the polar phase was dried for further chemical derivatization. It consisted of methoxyamination and the subsequent silylation, namely, by incubation with 40 µL of 40 mg/mL methoxyaminehydrochloride in pyridine for 90 min at 37 °C and an 80 µL silylation mixture containing 70 µL N,O-bis-(trimethylsilyl)-trifluoroacetamide (BSTFA) and 10 µL alkanes in pyridine for 30 min at 30 °C.

Gas chromatography (GC) was performed with a phenyl/polysiloxane (5:95%) coated 40 m capillary column with an inner diameter of 0.25 mm. The column included 10 m of a nonseparating precolumn. One µL of the final sample volume was injected. Mass spectrometry (MS) was performed at nominal mass resolution using an electron impact ionization time of flight mass spectrometer [36].

Data mining was conducted using TagFinder Software [37] after chromatogram-specific alkane-based retention index calculations were performed. The chromatograms were baseline corrected, and mass-fragment abundances were retrieved as chromatographic peak heights. Annotation was manually supervised using the Golm Metabolome Database Mass-spectral Collection (GMD, http://gmd.mpimp-golm.mpg.de/, accessed on 15 June 2018) [38]. At least three mass fragments per analyte were used within the TagFinder software for linearity and specificity checks and for relative quantification. Abundance data, i.e., the peak heights of these mass features were normalized to the internal standard for ^13^C_6_-sorbitol and the sample fresh weight. In the case of fungal supernatants, the abundance data were normalized to 300 µL instead of fresh weight.

### 2.4. Statistical Analysis of Metabolite Data

The normalized abundance data of the shoots, roots, fungal mycelia and fungal supernatant were separately analyzed. Missing value (NA) replacement was achieved using the *input.knn* function of the impute package [39] for the R environment. The normalized abundance of mass features representing each metabolite was divided by the averaged normalized abundance of the control treatment of each plant species to obtain the relative abundance ratios, i.e., fold changes relative to the noninoculated—optimal Pi control conditions. These ratios were subsequently log_2_-transformed. The log_2_-transformed ratios were used to compare the metabolic responses to each treatment combination. To determine the interaction between the “Pi status” (optimal/starvation) and “*Fsol* presence” (control/inoculated), experimental factors, two-way analysis of variance (two-way ANOVA) was applied to the combination of these treatments within each of the *Lotus* species (*p* < 0.05). Pairwise comparisons were assessed using the Student’s Test (*p* < 0.05). Only the mass features of the metabolites that were detected in at least 60% (before the NA replacement) of the experimental replications of each combination of the treatments were considered for the statistical analysis. The average values obtained from non-sample SAB medium samples were subtracted to estimate the quantity of secreted fungal metabolites in the cultivation supernatants. Principal component analysis (PCA) was performed after missing value substitution using the packages FactoMiner [40] and Factoextra [41] for the R environment.

### 2.5. Network Analysis of the Metabolite Data

To assess the patterns of correlation and the respective *Hub* metabolites in correlation networks, we conducted network analysis based on correlation matrices of Log_2_-transformed fold changes of normalized metabolite abundances. Instead of noninformative correlation across all combinations of experimental conditions, we analyzed the subsets of each treatment, namely, Control, FUS+, FUS+P- and P- of the shoot and root organs, and the two *Lotus* species separately. Pairwise Pearson´s correlation coefficients were calculated among all the metabolites of each of the subsets. To avoid the selection of an arbitrary numerical correlation threshold, we applied the context likelihood of relatedness algorithm (CLR) to define or reject a correlation between metabolites [42]. The CLR was applied to the complete correlation matrix to remove spurious correlations [43]. The calculation of correlations and CLR transformation were performed using the functions cor and clr of the stats and parmigene R-packages, respectively [44]. We constructed weighted permuted networks based on the resulting adjacency matrices of metabolites using the functions *graph_from_adjacency_matrix* (*weighted = TRUE*, *mode = “undirected”*, *diag = FALSE*) and *permute* (*sample (vcount)*) from the *igraph* R package [45]. This procedure resulted in the treatment-specific individual networks of each experimental condition, organ and species. We selected the *degree* of node (in the following degree) as a network topology parameter, which allowed us to select the nodes with the most interactions in the network. The degree of a node is defined by the number of edges linking it to other nodes within the network [46]. If the topology of a network contains few highly connected nodes, they are defined as *H*ubs [47]. In our current study, the most interactive nodes, i.e., the nodes with the highest degree values, represented the *H*ub metabolites of each experimental condition. We assigned relevance values to the *H*ub metabolites following previous studies that proposed and explored this concept [43,47]. As an additional parameter to characterize metabolites according to their position relative to the other metabolites of the network, we included *betweenness centrality* (in the following betweenness) calculations for each node. The betweenness value of a node is determined by the number of geodesic distances between the other two nodes in the network [46]. This parameter captures, in layman’s terms, the extent that a given node is “in-between” other nodes on paths across a network and thereby indicates the importance of a given node for connecting any two other nodes in a network. Betweenness is considered an indicator of the importance of a node for the potential flux of information between nodes of a network [48].

## 3. Results

### 3.1. Interaction between the Fusarium solani Endophyte and Lotus spp. under Extreme Pi Starvation

**Figure 1:**
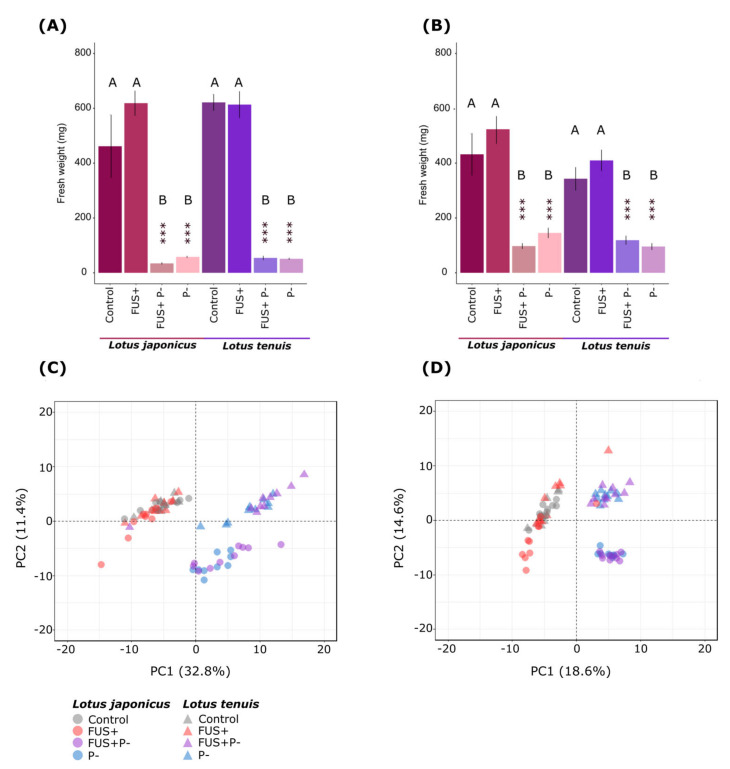
Modification of biomass of the shoots (**A**) and roots (**B**) of *L. japonicus* and *L. tenuis* after 32 days of inoculation with *F. solani 142L52B* and Pi starvation treatment. Statistical differences between the control condition (noninoculated—optimal Pi) and each treatment were assessed using Student´s test (mean ± standard deviation), *** *p* < 0.001. The different letters indicate statistically significant differences according to Tukey’s test (*p* < 0.05). Principal component analysis (PCA) based on the metabolite profiles of shoots (**C**) and roots (**D**) of *L. japonicus* and *L. tenuis* after 32 days of inoculation with *F. solani 142L52B* and Pi starvation treatment: Control (Non-Inoculated-Optimal Pi), FUS+ (inoculated—Optimal Pi), P- (non-inoculated—Pi starvation), FUS+P- (inoculated—Pi starvation). The relative concentration of metabolites references the *Control* condition and is log_2_-transformed.

To assess the involvement of the Pi status in the response of both *Lotus* species to inoculation with the endophyte *Fsol*, we analyzed the biomass after a growth period of 32 days, according to the time of the most evident differences between control and Pi starvation conditions, and before starting the flowering period. Pi starvation treatment significantly affected both *Lotus* species. Pi starvation reduced the biomass of shoots and roots, independent of the presence of *Fsol* (Figure 1A,B).

To assess the impact on central metabolism, we analyzed a metabolite fraction enriched for primary metabolites that contained 160 polar metabolites in the shoot analyses and 158 in roots [35,49,50]. In agreement with the effect on biomass, Pi starvation affected *the* primary metabolism as a dominant experimental factor, with only minor contributions of *Fsol* inoculation to the total variance of our data set (Figure 1B,C). In addition to the effects of experimental interventions, samples grouped according to the plant species were the second most important factor affecting primary metabolism in *Lotus* species, similar to earlier comparative metabolite profiling studies on abiotic stresses [35,49]. Multivariate analysis by PCA of shoot data revealed that PC1 explained 32.8% of the variance. PC1 grouped Pi-sufficient and Pi-starved samples irrespective of the presence of the endophyte (Figure 1C). The species difference and presence of species-specific responses to Pi starvation became apparent through PC2, with 11.4% of the variance explained. The PCA results of the root data displayed similar patterns to those detected for shoots (Figure 1D). In this case, the grouping induced by Pi starvation was more evident in PC2, with 14.6% of the variance explained, and the metabolic responses of the roots from the two species overlapped.

After statistical analysis, detailed differences between all treatments and the control condition became apparent, including FUS+ inoculation and the combination FUS+P-. The results of the statistical testing of all metabolites detected in the shoots and roots are reported in Supplementary Tables S1 and S2, respectively. In addition to statistically significant differences, we searched for metabolites that became nondetectable after *Fsol* treatment and/or due to Pi starvation. Conversely, we found metabolites that were absent in the control conditions and became detectable only in the P- and/or FUS+P- treatment conditions (Supplementary Tables S1 and S2). These metabolites were considered relevant without statistical testing and were scored as present or absent.

### 3.2. Phosphorylated Compounds

**Figure 2:**
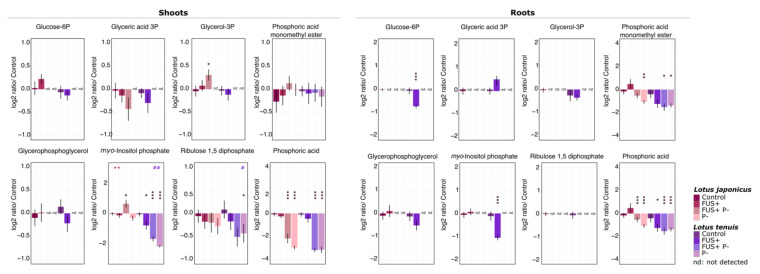
Log_2_ relative concentration of phosphorylated metabolites detected in the shoots and roots of *L. japonicus* and *L. tenuis* after 32 days of treatment. Control (noninoculated—optimal Pi), FUS+ (inoculated—optimal Pi), P- (noninoculated—Pi starvation) and FUS+P- (inoculated—Pi starvation). Statistical differences between the control (noninoculated—optimal Pi) and each treatment (FUS+, P- and FUS+P-) were assessed using Student´s test (mean ± standard deviation, *n* = 10), * *p* < 0.05, ** *p <* 0.01, *** *p* < 0.001, nd: not detected. Two-way ANOVA results of the interaction *FUS+P-*; ++ *p* < 0.01 for *L. japonicus*; # *p* < 0.05 and ## *p* < 0.01 for *L. tenuis*.

As expected, Pi starvation affected the phosphorylated metabolites in both plant species. In agreement with our PCA results, extreme Pi starvation had stronger effects on the root tissues than on shoots, with a clear decrease in and several absence scores for phosphorylated metabolites. The glucose-6-phosphate (Glu6P), glycerophosphoglycerol, glycerol-3-phosphate (glycerol-3P) and myo-inositol-phosphate (myo-inositol-P) levels dropped below the detection limits in the roots of both species under Pi starvation conditions (Figure 2; Supplementary Table S2).

In addition, *Fsol* inoculation of *L. tenuis* under optimal Pi supply caused a significant decrease in the concentration of phosphoric acid (Figure 2; Supplementary Table S2), while *L. japonicus* plants, under the influence of *Fsol*, maintained phosphoric acid levels similar to the control conditions. Two-way ANOVA (*p* < 0.05) applied to analyze further phosphorylated compounds detected in the roots was not significant (Supplementary Table S2).

Despite the decrease in Pi-related compounds in the roots, the concentration of phosphorylated metabolites in the shoots remained mostly unaffected according to pairwise statistical testing (Figure 2). However, the results obtained from two-way ANOVA revealed interactions between the Pi status (optimal/starvation) and *Fsol* presence (control/inoculated) experimental factors. Significant interactions were detected for the myo-inositol-P in *L. japonicus* and *L. tenuis* shoots and for ribulose 1,5-diphosphate (Ribulose 1,5-2P) in the *L. tenuis* shoots (Figure 2). The differences observed in both *Lotus* species for myo-inositol-P exhibited a statistically significant increase in the shoots of *L. japonicus* for the FUS+P- treatment, while the opposite effect was observed in *L. tenuis*.

### 3.3. Metabolites Representing Potential Carbon Sources

**Figure 3:**
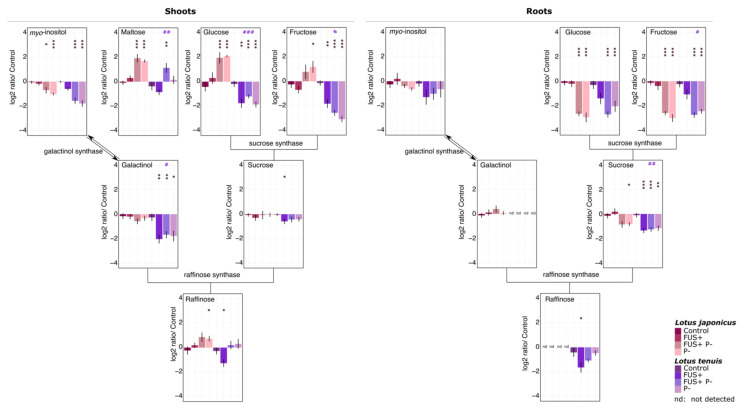
Log_2_-transformed relative concentration of sugars detected in the shoots and roots of *L. japonicus* and *L. tenuis* after 32 days of treatment: Control (noninoculated—optimal Pi), FUS+ (inoculated—optimal Pi), P- (noninoculated—Pi starvation) and FUS+P- (inoculated—Pi starvation). Statistical differences between the *Control* (noninoculated—optimal Pi) and each treatment (FUS+, P- and FUS+P-) were assessed using Student´s test (mean ± standard deviation, *n* = 10), * *p* < 0.05, ** *p <* 0.01, *** *p* < 0.001, nd: not detected. Two-way ANOVA results of the interaction *FUS+P-*; # *p* < 0.05, ## *p* < 0.01 and ### *p <* 0.001 for *L. tenuis*.

The partitioning of carbon resources is a key factor in plant–fungal interactions. We emphasize carbohydrate partition analysis by examining the potential carbon mobilization within the plants and the pathways involved in this process. Our results displayed differential responses regarding the sugar quantities after Pi starvation and *Fsol* inoculation. The concentration of sugars detected in the shoots and that detected in the roots are shown in Figure 3.

The results revealed changes in the sucrose levels. The amount of sucrose decreased significantly in the roots of *L. tenuis* for all the treatments evaluated. In contrast, *L. japonicus* responded with a significant decrease only after Pi starvation. Furthermore, fructose increased in the shoots of *L. japonicus* after Pi starvation, while in *L. tenuis,* fructose and glucose decreased slightly in response to the Pi starvation treatment. Moreover, *Fsol* elicited the reduction of fructose in *L. tenuis* under Pi-sufficient conditions*.* The shoots of *L. tenuis* exhibited significant interactions of the Pi status and *Fsol* experimental factors for four of the six sugars detected in our analysis. This interaction had an additive effect on the sugar levels (Figure 3; Supplementary Table S1).

We detected an increase in maltose levels in the treatments involving Pi starvation. Both plant species showed the same tendency of a major increase in maltose content in shoots for the FUS+P- treatment combination. This effect was strongest in *L. japonicus* and significant in the two-way ANOVA of *L. tenuis* (Figure 3).

The sugar conjugate galactinol, which is a biosynthesis intermediate of the raffinose family of sugars, was also detected by our analysis. The levels of galactinol decreased exclusively in the shoots of *L. tenuis* under all the treatment combinations (Figure 3). In addition, differences in the levels of raffinose were detected in both *Lotus* species. In *L. japonicus*, statistically significant differences were observed in the P- treatment when the levels of raffinose were increased, while in *L. tenuis,* the reduction of this sugar was observed in the FUS+ treatment (Figure 3).

In the shoots, α-α-trehalose was reduced in all the treatments of *L. tenuis*. In contrast, this sugar was detected in roots at increasing levels when inoculated with *Fsol*. The amount of α-α-trehalose was significantly higher in *L. japonicus* following inoculation with *Fsol*. In the case of *L. tenuis*, the increase in this sugar in the roots was only significant in the FUS+P- treatment (Supplementary Table S2).

**Figure 4:**
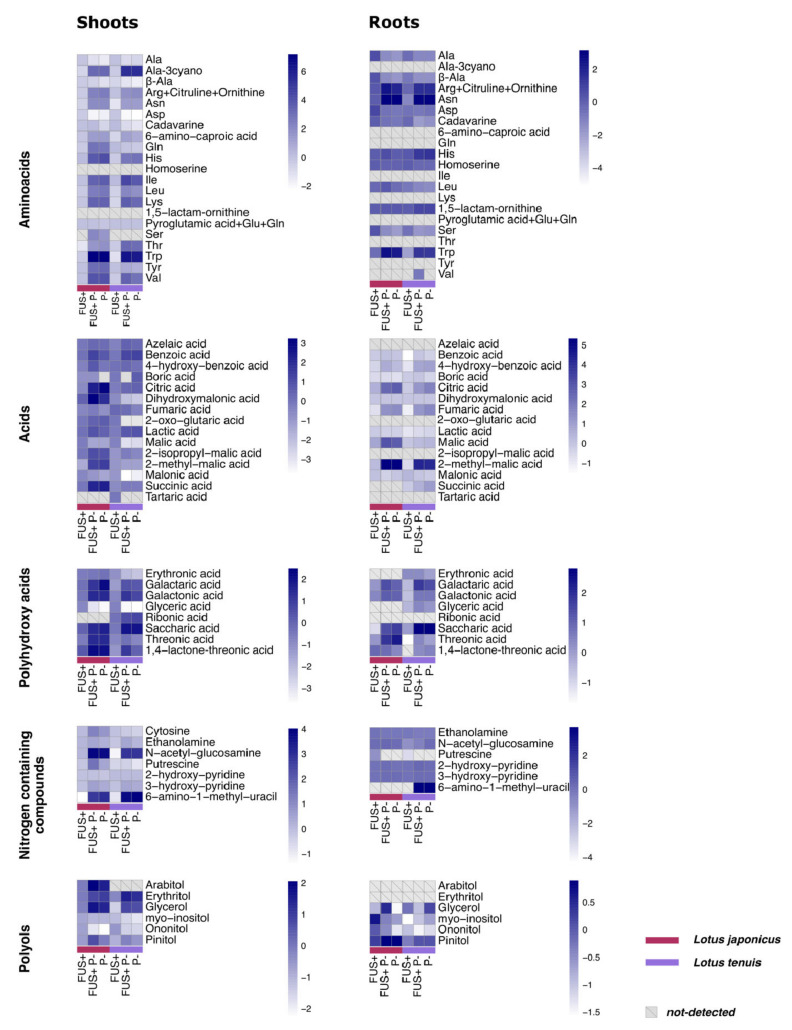
Log_2_-transformed relative concentration of metabolites detected in the shoots and roots of *L. japonicus* and *L. tenuis* after 32 days of treatment. FUS+ (inoculated—optimal Pi), P- (noninoculated—Pi starvation) and FUS+P- (inoculated—Pi starvation).

Polyols (also called sugar alcohols) exhibited a specific differential response according to the plant species and treatments. Arabitol, erythritol and glycerol accumulated in the shoots of both plant species under Pi starvation. On the other hand, the ononitol and myo-inositol levels decreased in both species under the same conditions. Ononitol and pinitol significantly decreased in the shoots of *L. tenuis* under the *Fsol* treatment. However, pinitol accumulation was detected in the roots of both plant species (Figure 4).

### 3.4. Organic Acids and Polyhydroxy Acids

The organic acid content belonging to the tricarboxylic acid pathway (TCA) can be modified under stress conditions, such as waterlogging [51], alkalinity [6] and salinity [49]. Modifications in the levels of these acids were detected in the treatments involving Pi starvation in both plant species.

The concentration of citric acid increased in the shoots and roots of *L. japonicus* under Pi starvation conditions, while in *L. tenuis*, this effect only occurred in roots. The 2-methyl malic acid responses were similar to that of citric acid (Figure 4). Succinic acid increased significantly in the shoots of *L. japonicus* under Pi starvation, while in *L. tenuis*, this compound increased only in the roots.

The glyceric acid and erythronic acid levels decreased under the FUS+P- and P- treatments in both *Lotus* species. The same effect was observed in *L. tenuis* for threonic acid. The rest of the detected polyhydroxy acids increased significantly under Pi starvation, e.g., saccharic acid, which is an intermediate compound in the TCA cycle (Figure 4; Supplementary Table S1).

### 3.5. Amino Acids and Other Nitrogen-Containing Compounds

Overall, the detected amino acids increased in both *Lotus* species and organs after treatment with Pi starvation (Figure 4; Supplementary Tables S1 and S2). Asparagine and tryptophan displayed the most relevant increases in the roots. These amino acids also showed interactions between our experimental factors, as detected by two-way ANOVA (Figure 4; Supplementary Table S2). Among other nitrogen-containing compounds, N-acetyl-glucosamine, 6-amino-1-methyl-uracil and 3-hydroxy-pyridine exhibited a statistically significant increase in the shoots of both *Lotus* species under the treatments involving Pi starvation. In the roots of *L. japonicus*, nitrogen-containing compounds were not detected or were not present in statistically significant amounts, except 3-hydroxypyridine. In the roots of *L. tenuis*, significant decreases in ethanolamine were detected for all the treatment combinations. A significant increase in 6-amino-1-methyl-uracil was detected in the roots of *L. tenuis* for the treatments involving Pi starvation (Figure 4, Supplementary Table S2). The results of two-way ANOVA applied to shoot measurements displayed a statistically significant interaction between factors for putrescine detected in the shoots of both *Lotus* species (Supplementary Table S1).

### 3.6. Metabolites of Mycelia and Secreted into the Medium by Fusarium solani

To detect the compounds that can be potentially secreted by the endophyte, we characterized the liquid supernatant medium after growing *Fsol* under in vitro conditions. The characterization of the primary metabolism of mycelial cells allowed the prediction of the compounds necessary for the normal growth of *Fsol* under optimal conditions.

**Figure 5:**
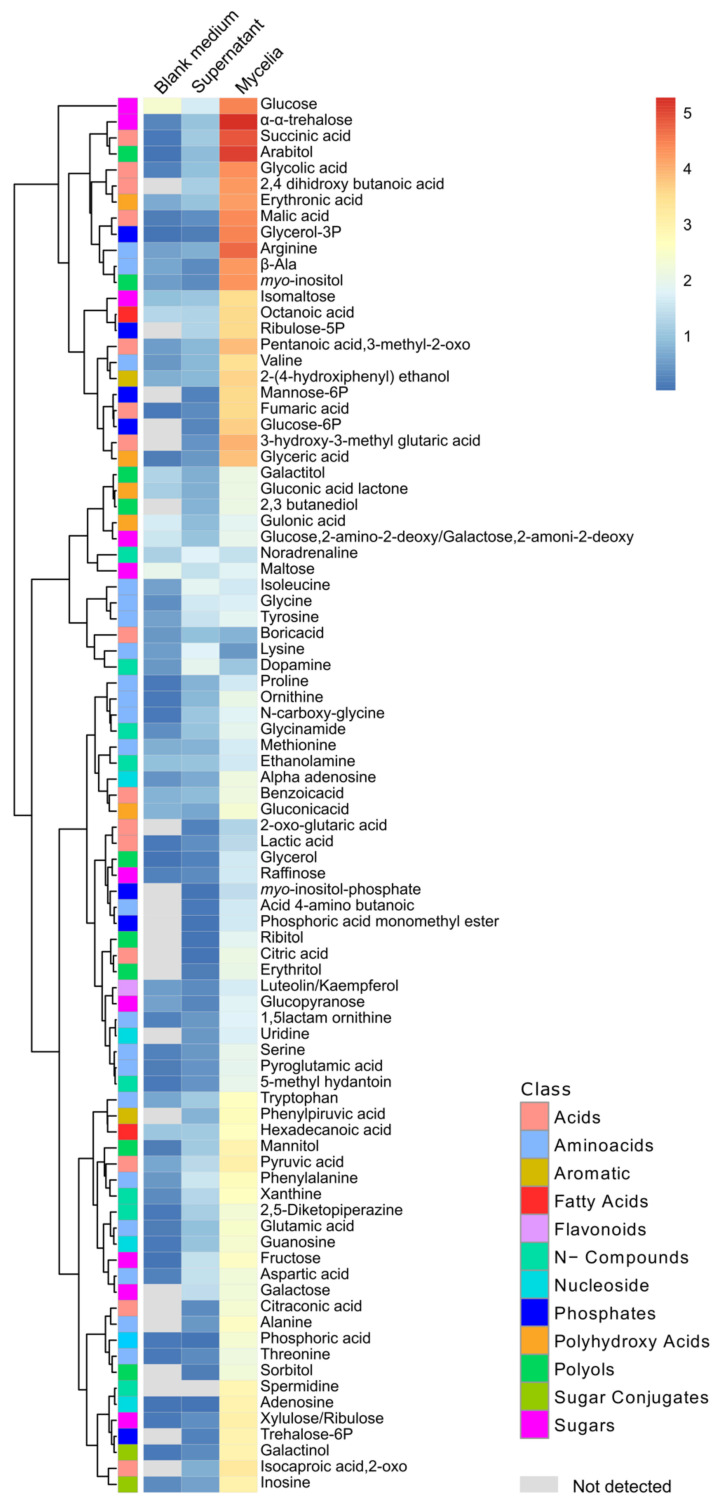
Relative concentration of metabolites detected in mycelia and the supernatant of *Fusarium solani 142L52B* after 7 days of incubation at 28 °C in Sabouraud broth (SAB). Relative concentration of metabolites expressed as the average of the normalized values to the median and corrected by non-sample controls (*n* = 5).

The results obtained in this analysis of metabolites produced by *Fsol* demonstrate that phosphorylated compounds were present mostly as intracellular components of the mycelia rather than secreted into the surrounding medium (Figure 5).

Glycerol-3P, glucose-6P and mannose-6-phosphate (mannose-6P) were the phosphorylated compounds with the highest apparent abundance detected in our analysis. These compounds were mainly detected in mycelium cells. Phosphoric acid and phosphoric acid monomethyl ester were present in mycelia at higher relative abundances than in the supernatant. In addition to other compounds, such as amino acids and polyols, myo-inositol and arabitol were detected in higher amounts in mycelium cells. As expected, the mycelia contained a higher amount of sugar than the supernatant medium (Figure 5). α,α-Trehalose was detected in high relative abundance in mycelia compared to the supernatant and blank medium. The relative abundance observed for this compound exceeded the values detected for other sugars in mycelia, such as glucose and isomaltose.

### 3.7. Correlation Network Analysis

To gain more insights into the changes in the metabolic network covered by our profiling analyses, we generated correlation networks of metabolite abundances across all replicate measurements of each of the 16 experimental conditions, namely, Control, FUS+, FUS+P- and P- treatments of shoots and roots from *L. japonicus* and *L. tenuis,* respectively. The resulting 16 correlation networks of metabolite nodes connected by edges representing CLR positive abundance correlations, generated as outlined in our “Material and Methods” section, were highly interconnected, as can be expected from metabolite profiling analyses enriched for primary metabolites (Supplementary Figure S1 and Table S3). The networks had similar overall shapes. Differences among the networks were difficult to detect and to recognize by visual inspection. Therefore, we performed an in-depth analysis of the network properties of degree and betweenness (refer to “Material and Methods” for definitions). We specifically identified *Hub* metabolites, i.e., those nodes in our networks that were most interconnected compared to average or typical nodes and thereby characterized the differences among the network topologies that were related to the investigated experimental conditions.

**Figure 6:**
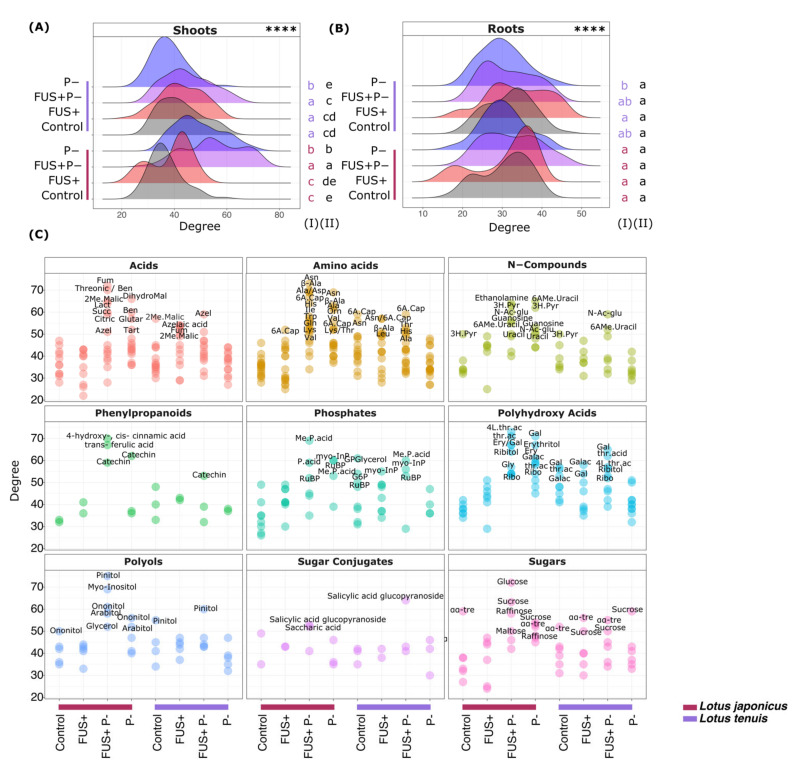
Density plot of the degree distribution calculated in each node of the correlation network analysis based on the pairwise Pearson-CLR-corrected correlations of log_2_-fold changes of metabolite concentrations detected in the shoots (**A**) and roots (**B**) of *L. japonicus* and *L. tenuis* after 32 days of treatment. **** Chi-square test of independence between distributions, *p* < 0.0001. Different letters to the right of (**A**) and (**B**) indicate statistically significant differences in the degree distributions according to Tukey’s test (*p* < 0.05) among the treatments of each species (I) and across all combinations of treatments and species (II). (**C**) Patterns of degree distribution of each metabolite classified according to the metabolic class. Control (non-inoculated—optimal Pi), FUS+ (inoculated—optimal Pi), P- (non-inoculated—Pi starvation) and FUS+P- (inoculated—Pi starvation), Fum: fumaric acid, Azel: azelaic acid, Ben: benzoic acid, Succ: succinic acid, Citric: citric acid, Lac: lactic acid, Tart: tartaric acid, Glutar: glutaric acid, DihydroMal: Di-hydroxy-malonic acid, 2Me.Malic: 2-methyl-malic acid, 2I.Malic.acid: 2-isopropyl-malic acid, 6A. Cap: 6-amino-caproic acid, Asn: asparagine, β-Ala: β-alanine, Ala: alanine, Asp: aspartic acid, His: histidine, Ile: isoleucine, Trp: tryptophan, Gln: glutamine, Lys: lysine, Val: valine, Orn: ornithine, Thr: threonine, Leu: leucine, 3H. Pyr: 3-hydroxy-pyridine, 6AMe. Uracil: 6-amino-1-methyl-uracil, N-ac-glu: N-Acetyl-glucosamine, Me.P.acid: phosphoric acid monomethyl ester, P.acid: phosphoric acid, RuBP: ribulose 1,5-2P, GPGlycerol: glycerophosphoglycerol, G6P: glucose-6P, MyoInP: myo-inositol-phosphate, Gal: galactonic acid, Galac: galactaric acid, Ribo: Ribonic acid, Gly: glyceric acid,Ery: erythronic acid, 4L.thr.ac: threonic acid-1,4-lactone, thr.ac: threonic acid, αα-tre: α-α-trehalose, “/” indicates “and”.

The degree distribution analysis of our correlation networks (Figure 6A,B) revealed degree ranges of ~20–60 or in part extending to >70 of the correlation networks of shoots (Figure 6A,C). In agreement with the smaller number of detected metabolites of the corresponding correlation networks of roots, the degrees ranged from ~15 to ~50 (Figure 6B).

The degree values did not follow normal distributions but were, in most cases, multimodal. Following visual inspection, multimodality was not found to be associated with our experimental conditions. Therefore, we neglected the multimodality aspect and focused on the clear shift of the degree distributions towards the higher values that we observed in the shoots of *L. japonicus*, specifically under the FUS+P- and P- treatments. In contrast to the other experimental conditions, multiple metabolites that we will term in the following as *Hub* metabolites passed an arbitrarily chosen degree threshold, i.e., >50, in the FUS+P- or P- *L. japonicus* shoot samples. (Figure 6A). None of the other degree distributions exhibited such clear shifts towards higher degree values except to a minor extent for the FUS+P- treatment of *L. tenuis* shoots. In this case, a small number of metabolites exceeded 50 degrees. Other than the correlation networks of shoot metabolites, no significant shifts of degree distributions that were associated with either treatments or species differences were observed for the root correlation networks (Figure 6B).

Motivated by the obviously altered network degree distributions associated with the treatment of Pi starvation in the absence of *Fsol* inoculation or in combination, we performed an in-depth analysis of the degree values of shoot metabolites. We highlighted global metabolite classes in this analysis, e.g., amino acids, and compared them across all eight metabolic correlation networks of the shoot samples (Figure 6C). *Hub* metabolites were present in all chosen metabolite classes, e.g., diverse organic or amino acids, nitrogen-containing compounds, phenylpropanoids, and diverse carbohydrates (Figure 6C). In general, and as expected from the degree distribution analysis, most *Hub* metabolites were present in the FUS+P- *L. japonicus* shoot network. Fewer *Hub* metabolites were present in the *L. tenuis* FUS+P- network or in the P- *L. japonicus* network. We found three main classes of metabolite observations; the first was the *Hub* metabolites (1) that had high degree values specifically in the FUS+P- *L. japonicus* shoot network. The second class was H*ub* metabolites (2) that were common to and highly ranked within the FUS+P- and P- *L. japonicus* networks. The FUS+P- *L. japonicus* shoot network yielded more *Hub* metabolites (Supplementary Table S3) and, in many cases, higher degrees than the respective P- network. The third class of *Hub* metabolites (3) had high degrees in the combinatorial stress FUS+P- of both the *L. japonicus* and *L. tenuis* shoot networks.

Among class (1), the specific H*ub* metabolites of the FUS+P- *L. japonicus* shoot samples were sugars and polyols. Glucose, sucrose and raffinose had high degree values in the *L. japonicus* FUS+P- network, whereas α-α-trehalose ranked highest in the respective control and in most of the other networks (Figure 6C). This observation was accompanied by high degree values for pinitol, ononitol, and myo-inositol in the *L. japonicus* FUS+P- shoot network. The pinitol degree value of 75 from this network was the largest overall observed degree value and was far higher than that in the respective *L. tenuis* FUS+P- network. Other FUS+P- *L. japonicus*-specific observations included phenylpropanoids, transferulic acid and 4-hydroxycinnamic acid, fumaric acid or, to a lesser degree, succinic acid.

Concerning class (2), i.e., the commonly high rankings in FUS+P- and P- *L. japonicus* H*ub* metabolites, we found several nitrogen-containing compounds and amino acids, for example, Asp and β-Ala. These compounds had the highest degree values in the *L. japonicus* FUS+P- and P- networks compared to 6-aminocaproic acid, which ranked highest in most of the other networks (Figure 6C). This class contained diverse metabolites, such as catechin, benzoic acid and several polyhydroxy acids, e.g., erythronic or galactonic acid.

Observation class (3) contained a set of biotic or abiotic stress-related compounds that had high degree values in the combinatorial FUS+P- stress networks. Salicylic acid glucopyranoside had the highest degrees in the *L. japonicus* and *L. tenuis* FUS+P- networks (Figure 6C). Azelaic acid ranked highest in the *L. tenuis* FUS+P- network and, similar to 2-methyl malic acid, was a *Hub* metabolite in the *L. japonicus* FUS+P-, *L. tenuis* FUS+, and *L. tenuis* Control networks. Catechin was a common H*ub* metabolite of the *L. japonicus* and *L tenuis* FUS+P- networks and overlapped with class (2) featured in the *L. japonicus* P- network. Catechin shared this pattern with, for example, N-acetyl-glucosamine, galactonic acid, threonic acid or phosphoric acid mono-methyl-ester (Figure 6C).

To further explore and specify the multiple observed H*ub* metabolites in our correlation networks of shoot samples, we calculated the betweenness centrality of a node as a second topology parameter. The betweenness of metabolite nodes was independent of the degree (Supplementary Figure S2). As specified in our “Materials and Methods” Section 2, betweenness can be considered an alternative indicator of the importance of a node for a potential flux of information between distant nodes within a network [48]. We assigned high relevance to metabolites with a combination of high degree and betweenness values and metabolites with high betweenness and low to medium degrees. Guided by the previous analyses, we focused on the combinatorial FUS+P- stress of the *L. japonicus* (Supplementary Figure S2A) shoot samples compared to the *L. tenuis* shoot samples (Supplementary Figure S2B).

For the correlation network of the combinatorial FUS+P- treatment of *L. japonicus,* 10 of 13 metabolites yielded high betweenness values, which were arbitrarily set as larger than the 95% quantile (Supplementary Figure S2A). This global result underlined the difference in the metabolic combinatorial stress state compared to single stress conditions. A set of *H*ub metabolites reported earlier for the combinatorial stress state had high betweenness values, namely, β–Ala, benzoic acid, myo-inositol, arabitol, ononitol galactaric acid, phosphoric acid and sucrose (Supplementary Figure S2A).

Applying the same betweenness threshold to the *L. tenuis* correlation networks, we discovered three metabolites with high betweenness, i.e., salicylic acid glucopyranoside, β–Ala, and ribonic acid (Supplementary Figure S2B). In agreement with its link to biotic stress responses, salicylic acid glucopyranoside had high betweenness in the *L. tenuis* FUS+ and FUS+P- networks but a high degree only under combinatorial stress. β–Ala had high betweenness and degree values only under FUS+ single stress conditions, and ribonic acid had high degree values under all stress conditions but increased degree values from FUS+ to P- and FUS+P- stresses (Supplementary Figure S2B).

## 4. Discussion

### 4.1. Pi Starvation Masks the Effect of the Endophyte Fusarium solani in Both Lotus Species

The results obtained in this study demonstrated that extreme Pi starvation in *Lotus* spp. constitute a stronger stress than the effect caused by the interaction with *Fsol*. The measurement of biomass performed in the experiment exhibited a morphological response to the combination of both treatments (FUS+ and P-) and displayed the same effect as the response produced by the effect of Pi starvation itself (P-). According to this result, the hypothesis that Pi constitutes a determinant factor in the interaction between *Fsol* and *Lotus* species is not supported. Although for biomass no differences were observed between the P- and FUS+P- treatments, particular variations in the levels of metabolites exposed differences in the responses by the two *Lotus* species to nutritional stress mediated by the presence of *Fsol* in combination with Pi starvation. The multivariate approach based on PCA indicated that roots were affected by Pi starvation, independent of plant species and *Fsol* presence. On the other hand, shoots revealed a differential response to Pi starvation according to the *Lotus* species. The Pi deprivation effect has been found to be related to a complex reprogramming of metabolism [52]. This assumption, in addition to the observations obtained in this work, could indicate that particular outcomes in response to Pi starvation cannot be fully explained by observing only biomass.

Roots were certainly the first organ affected by the nutritional stress evaluated. In this trend, the information obtained from the metabolomics analysis shows that Pi depletion is evident in the root system. However, shoots conserve a certain concentration of phosphorylated metabolites to maintain metabolic functions.

The biomass production response suggests that extreme Pi starvation might be a minor aspect of the differential behavior of the interactions between *Lotus* spp. and *Fsol* observed in our previous study. Moreover, different patterns observed in the phosphorylated compounds in the correlation network indicate the possibility that both *Lotus* species have different mechanisms to deal with Pi starvation, independent of the presence of *Fsol*.

### 4.2. Modulation of Carbohydrate Availability by Lotus spp.-Fusarium solani Interactions under Pi Starvation Varies According to the Lotus Specie

In addition to the differential response observed in phosphorylated compounds, we also detected differential behaviors in the response related to the sugar content in *L. japonicus* and *L. tenuis* under Pi starvation. Our results show an increase in sugar compounds as a consequence of Pi deprivation. Nevertheless, this effect was only observed in *L. japonicus*, while *L. tenuis* exhibits decreases in the levels of glucose and fructose under the same stress conditions.

The induced carbohydrate shortage extends to the invertase-cleavage products of sucrose. Pi starvation induces a reduction in glucose/fructose that is highly similar in *L. tenuis* compared to *L. japonicus* roots. However, *L. japonicus* accumulated glucose in shoots in response to the Pi limitation. This result clearly demonstrated the differential carbohydrate availability in *L. japonicus* compared to *L. tenuis*. Taken together with sucrose observations, *L. tenuis* appears to become limited in soluble carbohydrates after inoculation with *Fsol* and Pi starvation. Furthermore, *L. tenuis* may not be able to control and limit carbohydrate/carbon withdrawal by *Fsol*. Thus, *Fsol* might be on the verge of being a mild pathogen of *L. tenuis*. This result is consistent with our previous study of the interaction of *Fsol-Lotus* spp., which demonstrated the differences in the carbohydrate contents of the two *Lotus* species after infection by *Fsol* under optimal growth conditions [14].

The raffinose pathway may support the observation of *Fsol*-induced sucrose and hexose shortages in *L. tenuis*. This pathway is also linked to galactose (conjugated from galactinol) and myo-inositol. Myo-inositol approximately mirrors the sucrose and raffinose concentrations in *L. tenuis* roots and the Pi levels in shoots. Indeed, raffinose was reduced in *L. tenuis* after *Fsol* inoculation but, like glucose, increased in *L. japonicus* as part of the Pi limitation stress response. This observation confirms the *Fsol*-induced sucrose shortage in *L. tenuis*. The galactinol levels also mirror sucrose reduction and may indicate a limitation of the galactinol precursors after *Fsol* inoculation of *L. tenuis*. In fact, galactinol reduction is more significant than raffinose reduction and part of the Pi limitation response. In summary, the effects observed in the raffinose biosynthesis pathway support the hypothesis that *Fsol* induces sucrose shortage in shoots and roots and glucose/fructose shortage predominantly in shoots. Both sucrose shortage and reduced hexose availability appear to limit galactinol/raffinose biosynthesis.

Maltose levels increased under Pi starvation. This result could be related to the transitory starch breakdown to supply the energetic requirements induced by the stress condition [53]. This effect was more evident when *Fsol* was present in combination with Pi starvation and was significant for the FUS+P- interaction in *L*. *tenuis*. Moreover, the levels of maltose in *L. tenuis* increase as a consequence of the additive effect of *Fsol* and Pi starvation. This result might reinforce the assumption regarding the hexoses requirement in the interaction between *Fsol* and *L. tenuis*.

The levels of α-α-trehalose decreased significantly in *L. tenuis* under the treatments involving *Fsol*, Pi starvation and the combination of both conditions. This compound has previously been described as a signaling molecule in abiotic stresses and plant microbial interactions [54]. Rhizobia–legume interactions analyzed in *Phaseolus vulgaris* demonstrated that an exogenous application of trehalose induces sucrose synthase and alkaline invertase activities [55]. This evidence reinforces the hypothesis of a sucrose shortage in *L. tenuis* in interaction with *Fsol*. In addition, this sugar was detected at a high level as a mycelium component.

It is relevant to note that the increase in the levels of α-α-trehalose in the roots of *Lotus* spp. is likely due to the presence of the endophyte *Fsol*. These results suggest that α-α-trehalose could be considered a potential indicator of *Fsol* presence in the root system. In addition, this sugar is highly related to important fungal functions. Trehalose has been described as a reserve carbohydrate and stress-related metabolite in yeast and filamentous fungi [56]. Moreover, the accumulation of trehalose by yeast under Pi starvation has also been described [57]. It also plays important roles in relevant biological traits, such as sporulation and infectivity of certain fungal pathogens [58], including *Fusarium* [59].

Sugars constitute more than photosynthesis-derived products. Different studies reported to date have demonstrated the role of sugars as signaling compounds [60,61,62,63]. From a nutritional point of view, it has been demonstrated that the Pi levels influence the sugar response [29]. The concentration of sugars increases under low Pi levels and enhances the expression of genes involved in the Pi starvation response [64]. Here, we observed an increase in sugar content only in *L. japonicus*. According to our results, *L. japonicus* may exhibit better performance than *L. tenuis* against Pi depletion. However, *L. tenuis* grows and successfully develops in restricted environments that have diverse abiotic stresses, including Pi limitation. Taken together, the evidence, in addition to our results, elucidates the occurrence of differential responses of both *Lotus* species to the same stress. In this trend, if sugars are able to act as signaling compounds in *L. japonicus*, it might not be the same mechanism used by *L. tenuis* to achieve the same response to the nutritional stress.

### 4.3. Nitrogen-Related Compounds Responded Differentially to Pi Starvation

In legumes, Pi starvation involves a cost of carbon and nitrogen in the root systems [65]. Pi starvation in legumes affects the utilization of organic nitrogen sources and the availability of organic carbon required for the synthesis of amino acids in nodules [66]. Despite the absence of nodulation under our experimental conditions, we detected differences in the content of amino acids provoked by Pi starvation.

We detected a significant increase in asparagine in the roots of both *Lotus* species under the effect of extreme Pi starvation. This response was also observed previously by Almeida et al. [67] in the roots of *Trifolium repens* under severe Pi starvation. Previous studies have stated that the synthesis of this amino acid can be induced by mineral deficiencies [66]. Similar behavior was observed in the arginine levels. The enhancement in the biosynthesis of arginine has also been described in legumes under nutritional stress involving Pi [68].

We observed an increase in tryptophan under Pi starvation. The concentration of tryptophan in the roots and shoots of both plant species might lead to the production of indole acetic acid (IAA). This effect has been correlated with the promotion of root growth, which triggers modification of the root architecture to improve nutrient acquisition [7,8,69,70,71]. Moreover, auxin and peptides have been described as regulatory components in signaling to regulate Pi acquisition. The pathways involving the synthesis of tryptophan have been associated with the production of the secondary metabolites and proteins necessary to address the shortage of amino acids [72]. Nevertheless, our results demonstrated an increase in the amount of tryptophan, but instead of shortage, we detected an increase in the levels of the other amino acids. However, only the involvement of asparagine with the rest of the metabolites was evident in the correlation network analysis. This result emphasizes the role of asparagine in the response to extreme Pi starvation and underlines the differential response between the model (*L. japonicus*) and the crop (*L. tenuis*).

The accumulation of polyamines has been correlated with the tolerance of *L. tenuis* and *L. japonicus* to abiotic stress [73,74,75,76]. However, the response of polyamines to different stress conditions in *Lotus* spp. seems to be dependent on the type of stress. Despite the relationship between polyamines and stress tolerance, it has also been demonstrated that the putrescine content is not in agreement with the saline stress in *L. tenuis* [9]. In our analysis, the content of putrescine, instead of accumulating, decreased in the roots of *L. tenuis* and shoots of *L. japonicus* under the Pi starvation treatment. Nevertheless, we observed an increase in putrescine in the shoots of *L. japonicus* under the combination of Pi starvation and the *Fsol* effect. Similar behavior was observed in the symbiosis between *L. tenuis* and mycorrhizal fungi under salinity stress. Echeverria et al. [76] stated that polyamine content is affected by the symbiosis between *L. tenuis* and *Rhizophaguis irregularis* (ex. *Glomus intraradices*) only when salt stress is present. This result added to the evidence observed in previous studies aimed at determining the multiple roles of polyamines in different biotic and abiotic stress conditions.

### 4.4. Organic Acids Might Play an Important Role in the Response to Pi Starvation

Elevated concentrations of organic acids belonging to the TCA cycle were exhibited in the treatments involving Pi starvation. An increase in succinic acid under Pi starvation was previously observed in the common bean by Hernandez et al. [77]. Nevertheless, the results obtained in the common bean differ from our results regarding other acids. We observed an increase in malic acid and citric acid in *L. japonicus* mediated by Pi starvation. This effect has been described before in *Brassica napus* [78,79], rice [80] and legumes such as *Virgilia divaricata* [81], chickpea [82] and lupine [83]. Moreover, Touhami et al. [84] demonstrated that forage legumes showed higher concentrations of the organic anions used to improve the acquisition of Pi in the root system than nonlegume grasses. The increase in the concentration of organic acids in roots has been related to the secretion of root exudates containing anions to solubilize phosphoric resources [85,86].

The metabolite correlation network analysis showed that fumaric acid had the highest number of correlations with other metabolites in *L. japonicus* for the treatment combining *Fsol* presence and Pi starvation. This result suggests fumaric acid as a potential *Hub* metabolite in the interaction between *Fsol* and *L. japonicus* under the effect of Pi starvation, revealing the possible role of fumaric acid in the regulation of the TCA cycle under these experimental conditions. Additionally, the role of this acid as a source of carbon transport could be considered [87].

### 4.5. Role of Polyols in the Pi Starvation Response

Our results showed an increase in the amounts of some polyols as a consequence of Pi starvation, such as arabitol, erythritol and glycerol. The accumulation of ononitol has been related to other abiotic stresses in *Lotus* spp. [49]. Nevertheless, in our study, the amount of this polyol was significantly reduced under Pi starvation conditions, with a further effect on the accumulation of pinitol. In this trend, it seems unlikely that ononitol is a metabolite representative of all abiotic stresses.

The multiple roles of polyol compounds have been previously reported as osmoprotectant molecules. However, their importance in the stress response has also been related to their ability to react as a quencher of reactive oxygen species [88]. Moreover, the physiochemical properties of polyols influence photosynthesis, respiration and developmental processes because they are a sink of photosynthates [89]. Polyols are an important part of photosynthesis-derived compounds, and they are transported as storage molecules via phloem [89]. Our results demonstrated the accumulation of some sugar alcohols under Pi starvation, while the concentrations of ononitol and pinitol decreased under the same conditions. This effect could be explained by their synthetic pathway, which is dependent on the concentration of Glu6P as the main precursor and is strongly affected by Pi starvation.

Polyols also accumulate in fungi for multiple functions, such as carbohydrate storage, osmoregulation, translocation of compounds and enzyme regulation [90]. These compounds were detected as mycelium components of *Fsol.* This observation supports the role of these compounds in fungal metabolism. In this trend, the variability in the number of polyols detected in roots could be partially explained by the presence of endophytes.

### 4.6. Phosphorylated Compounds Are Accumulated Instead of Excreted by Fusarium solani

The results obtained in the metabolic analysis of mycelia and supernatant medium after the growth of *Fsol* demonstrated that phosphorylated compounds are constitutive metabolites of the mycelia rather than excreted in the medium. Because phosphorus is a limiting nutrient in the production of secondary metabolites by fungi [91], its function in the metabolism of *Fsol* is relevant. In addition, the metabolic profile obtained from *Fsol* showed the accumulation of phosphoric acid in the mycelia in values corresponding to a ratio of 0.5 of glucose. This assumption might suggest competition between *Lotus* plants and *Fsol* for Pi resources under extreme Pi starvation. However, our results demonstrate neither amelioration nor declining Pi stress under the effect of *Fsol*. This premise is relevant considering that *Fsol* was isolated from the roots of *L. tenuis* growing in soils with low Pi availability [14].

### 4.7. Correlation Network Analysis Remarks

The degree patterns in the control conditions displayed lower degree values than those obtained in the different treatments involving *Fsol* and Pi starvation in both *Lotus* species. This effect could be interpreted as an equilibrium under control conditions, mediated primarily by the behavior of pathways under optimal conditions. On the other hand, an increase in the degree values for the different treatments evaluated indicates specific metabolites that vary similarly to the stress conditions. This effect was also observed in the rise or impairment of specific metabolites absent in control conditions. The detection values were observed only in the FUS+, P- or FUS+P- conditions.

Network analysis showed the relevance of pinitol in the involvement with the other metabolites analyzed. This effect, observed only in *L. japonicus* for the combination of *Fsol* and Pi starvation, denotes the differential mechanisms of response to the Pi stress combination for both *Lotus* species. Our results also exposed the presence of myo-inositol and other alcohol sugars as metabolites that can be determinant in response to the combination Pi starvation *Fsol.* In addition, the involvement of sugars and sugar conjugates in the metabolic response to FUS+P- was confirmed in the analysis of topology parameters derived from the correlation network analysis. The presence of indicators of the involvement of polyols and sugars resulting from the observations obtained from the degree and betweenness centrality analyses reinforces the hypothesis regarding the sugar shortage in *L. tenuis* mediated by *Fsol*.

The relationship between pinitol and myo-inositol displays the involvement of polyols and sugar pathways in the interaction between *L. japonicus*–*Fsol*–Pi starvation. These compounds may be the origin of new hypotheses and experiments to perform a deep characterization of the pathways involved in the synthesis, storage and translocation of carbon substrates. In addition, the involvement of fumaric acid in this system reveals potential alternative pathways to address the energy demand produced by the combination of Pi starvation and *Fsol* presence.

## 5. Conclusions

*F. solani* cannot modify the effect of extreme nutritional stress. However, the effects observed in carbon mobilization depend on the *Lotus* species and might be facilitated by the interaction with *Fsol*. The presence of several *Hub* metabolites involved in the combination of *Fsol* and Pi starvation exposed the complex outcome caused by *F. solani* on the metabolism of *L. japonicus* under this condition. Among our results, sugars and sugar-related compounds exhibited relevant alterations in the metabolic response to the FUS+P- combination. A deeper focus on the mechanisms underlying these particular interactions would provide information that allows the performance of plant–fungal interactions in constrained environments to be predicted.

## Data Availability

The data presented in this study are available on request from the corresponding author.

## Supplementary Materials

The following are available online at https://www.mdpi.com/article/10.3390/jof7090765/s1, Figure S1: Network plots based on the pairwise Pearson-CLR-corrected correlations of Log_2_-fold changes of metabolite relative concentrations, Figure S2: Scatter plot of the degree and betweenness centrality parameters obtained from the networks based on pairwise Pearson-CLR-corrected correlations of Log_2_-fold changes of metabolite relative concentrations for shoots (A) and roots (B), Table S1: Results of two-way ANOVA and Student´s test applied to metabolites detected in shoots, Table S2: Results of two-way ANOVA and Student´s test applied to metabolites detected in roots, Table S3: Topological parameters of each correlation network.
