# Supplementary material for: Metabolic Profiling and Metabolite Correlation Network Analysis Reveal That Fusarium solani Induces Differential Metabolic Responses in Lotus japonicus and Lotus tenuis against Severe Phosphate Starvation
Source: J Fungi (Basel). 2021 Sep 16;7(9):765. doi: 10.3390/jof7090765 (PMC8468338; doi:10.3390/jof7090765)
Supplement: Supplementary file 1 [file jof-07-00765-s001.zip › Supplemental Files/Supplemental Table 3.pdf]

Supplemental Table 3. Topological parameters for each correlation network based on pairwise Pearson-CLR-corrected correlations of Log2 fold change concentration of metabolites detected in shoots and roots of L. japonicus and L. tenuis after 32 days of the following treatments: Control (Non-inoculated - Optimal Pi), FUS+ (Inoculated - Optimal Pi), P- (Non-inoculated – Pi starvation) and FUS+ P- (Inoculated - Pi starvation).

Network			Degree (media ±SD)	Edge number	Node number	Network diameter	Edge conectivity	Edge density	Node conectivity
Shoots	*L. japonicus*	Control	36.97±6.47	1201	65	0.091	27	0.577	27
		FUS+	37.75±8.63	1189	63	0.074	22	0.608	22
		FUS+P-	52.30±10.61	1987	76	0.069	29	0.697	29
		P-	46.62±8.65	1681	72	0.039	33	0.657	33
	L. tenuis	Control	42.38±8.01	1441	68	0.043	30	0.632	30
		FUS+	42.78±8.18	1433	67	0.032	22	0.648	22
		FUS+P-	45.04±8.99	1599	71	0.047	29	0.643	29
		P-	37.86±6.16	1306	69	0.079	27	0.556	27
Roots	*L. japonicus*	Control	30.70±6.08	660	43	0.125	17	0.730	17
		FUS+	31.30±7.45	720	46	0.138	15	0.695	15
		FUS+P-	32.32±7.09	808	50	0.060	19	0.659	19
		P-	30.35±5.58	789	52	0.136	17	0.595	18
	L. tenuis	Control	31.82±5.74	875	55	0.049	20	0.589	20
		FUS+	33.88±7.44	847	50	0.091	18	0.691	18
		FUS+P-	31.49±6.38	803	51	0.077	21	0.629	21
		P-	30.00±5.50	750	50	0.056	20	0.612	20
